# Preparation, Characterization, and Anti‐Fatigue Activity of Clamworm (*Perinereis aibuhitensis*) Hydrolysate and Its Peptide and Polysaccharide Fractions

**DOI:** 10.1002/fsn3.72319

**Published:** 2026-11-11

**Authors:** Dan Qiu, Songran Fang, Fengying Cui, Honglei Zhou, Meichao Zhang, Zifang Zhao, Wenhai She, Adewale Olusegun Obadina, Haohao Wu, Wei Wang

**Affiliations:** ^1^ Department of Pathology The Affiliated Hospital of Qingdao University Qingdao China; ^2^ State Key Laboratory of Marine Food Processing & Safety Control, College of Food Science and Engineering/Sanya Oceanographic Institution Ocean University of China Qingdao/Sanya China; ^3^ Fujian Yangze Marine Biotechnology Co., Ltd Fuzhou China; ^4^ Weihai Institute for Food and Drug Control Weihai China; ^5^ Hainan/Haikou Research & Development Center for Biopeptide Engineering Huayan Collagen Technology Co., Ltd. Haikou China; ^6^ Guangdong Aquatic Resources Industrialization Engineering Technology Research Center Guangzhou Luxe Seafood Enterprises Ltd. Guangzhou China; ^7^ Department of Food Science and Technology Federal University of Agriculture Abeokuta Nigeria; ^8^ Department of Biotechnology and Food Technology University of Johannesburg Johannesburg South Africa

**Keywords:** BCAAs, clamworm hydrolysate, energy metabolism, exercise performance, peptides, polysaccharides

## Abstract

Fatigue is a prevalent health issue necessitating effective nutritional interventions. *Perinereis aibuhitensis* (clamworm) is a commercially important marine species traditionally valued for its rich nutritional profile and medicinal properties. This study investigated the preparation, characterization, and anti‐fatigue activity of *Perinereis aibuhitensis* hydrolysate (PAH). Optimized enzymatic hydrolysis yielded PAH rich in small molecular peptides (< 1000 Da, accounting for 77.9% of total peptide) with abundant branched‐chain amino acids (12.28 g/100 g), as well as polysaccharide fractions tentatively characterized as putative glycogen‐like glucans and sulfated heteroglycans. In male C57BL/6 mice, PAH and its fractions (PAT, peptides; PAS, polysaccharides) significantly enhanced exercise endurance in exhaustive swimming, extending swimming time by 83.23%–114.52% compared with the control (*p* < 0.05). Furthermore, PAH effectively maintained muscle morphology, boosted energy reserves (glycogen and ATP), reduced metabolite accumulation (lactic acid, urea nitrogen, and creatine kinase), and enhanced antioxidant enzyme activities (superoxide dismutase and glutathione peroxidase increased by 42.92%–65.34% and 42.51%–52.49%, respectively). These findings reveal that the potent anti‐fatigue efficacy of PAH may be associated with the contributions of both its bioactive constituents of BCAAs, putative glycogen‐like polysaccharides, and sulfated polysaccharides. This study provides a theoretical basis for developing PAH as a promising anti‐fatigue dietary supplement.

## Graphical abstract

*Perinereis aibuhitensis* hydrolysate (PAH), rich in BCAA‐peptides and polysaccharides, significantly enhances mouse swim endurance (+83.23%–114.52%) by boosting energy reserves (glycogen/ATP), clearing metabolites, and upregulating antioxidant enzymes, supporting its potential as an anti‐fatigue dietary supplement.

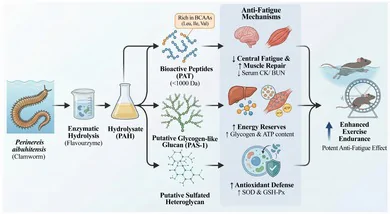

## 1 Introduction

The rapid development of modern society has made fatigue a common physiological condition (Lim et al. 2020). Beyond compromising life quality and work performance, fatigue serves as a key risk factor for various chronic diseases, including Parkinson's disease, multiple sclerosis, and immune deficiency syndromes (Lei et al. 2024; Luo et al. 2019). The etiology of fatigue involves multifaceted mechanisms, principally characterized by “energy exhaustion” and “oxidative surge” (Cai et al. 2024). The former refers to the depletion of energetic substrates (e.g., adenosine triphosphate [ATP]) and accumulation of metabolites (e.g., blood lactic acid, blood urea nitrogen) under sustained physical exertion (Wang, Wang, et al. 2021); while the latter involves oxidative stress damage caused by altered antioxidant enzyme activities (e.g., superoxide dismutase [SOD]) and massive reactive oxygen species (ROS) production, jointly resulting in mitochondrial dysfunction, diminished muscle contractility, and impaired motor performance (Wang et al. 2020; Yan et al. 2024). Although pharmacological interventions such as antidepressants and stimulants are commonly used to alleviate fatigue, their long‐term administration is often associated with adverse cardiovascular and neurological side effects (Li, Zhu, et al. 2022). Hence, developing safe and effective anti‐fatigue functional foods is of great importance.

Marine biological resources, abundant in unique bioactive compounds, are increasingly recognized as valuable reservoirs for discovering novel anti‐fatigue agents. Recent studies have demonstrated that marine‐derived bioactive substances, particularly functional peptides and polysaccharides, can effectively facilitate physiological recovery through distinct molecular mechanisms. For instance, active peptides derived from *Auxis thazard* and sea cucumber have been shown to maintain energy homeostasis and enhance exercise performance by modulating the AMPK/PGC‐1α signaling pathway (Qu et al. 2022; Wang, Shi, et al. 2021). Similarly, salmon skin collagen peptides can alleviate fatigue by enhancing mitochondrial biogenesis and reducing oxidative stress (Ma et al. 2025). Furthermore, marine polysaccharides and glycoproteins, such as those from *Spirulina platensis* and hairtail by‐products, exhibit potent anti‐fatigue effects by regulating central nervous system mechanisms or boosting antioxidant enzyme activities (Chen et al. 2023). Other marine hydrolysates, including those from oyster, monkfish liver, and *Lateolabrax japonicus* , have also been validated to exert significant fatigue‐relieving effects, providing a broad basis for marine functional food development (Li, Li, et al. 2024; Miao et al. 2018; Xu et al. 2017).

Clamworm (*Perinereis aibuhitensis*, PA) is a key economic aquaculture species and an important feed source for fish, shrimp, and crabs (Sun et al. 2019). It is rich in high‐quality proteins, essential amino acids, polysaccharides, and other bioactive compounds, conferring substantial nutritional value (Nie et al. 2025; Teng et al. 2025). Traditionally, as recorded in the Chinese pharmacopeia *Compendium of Materia Medica*, clamworm has been used to treat various ailments, highlighting its potential in functional food development. Existing studies have demonstrated that bioactive extracts from clamworm exhibit multiple physiological activities, including antioxidant, immunomodulatory, anti‐inflammatory, antithrombotic, and antitumor effects. For instance, Li et al. (2017) identified polysaccharides in a decoction‐based extract of clamworm and demonstrated its favorable antithrombotic activity through the inhibition of thrombin and fibrinogen. Similarly, Liu et al. (2022) prepared a clamworm hydrolysate via enzymatic hydrolysis and confirmed their antioxidant activity. Furthermore, Zhang et al. (2022) characterized oligopeptides isolated from clamworm hydrolysate, revealing their ability to inhibit H1299 cell proliferation and induce apoptosis, thereby exerting antitumor effects. These findings collectively demonstrate the potential of clamworm extracts as functional ingredients.

The specific potential of clamworm hydrolysate to enhance exercise performance remains largely unexplored. Although its antioxidant capacity has been verified (Liu et al. 2020), whether this translates to an effective anti‐fatigue capability in vivo has not yet been elucidated. This knowledge gap hinders the high‐value utilization of clamworm resources. Given the compositional complexity of clamworm hydrolysate, its health benefits are likely not attributed to a single component acting in isolation, but rather derive from the respective contributions of multiple active constituents. Numerous studies have reported the anti‐fatigue properties of marine‐derived hydrolysates and polysaccharides. However, most existing investigations have focused solely on the crude hydrolysate or a single bioactive fraction from a given organism. The simultaneous isolation, evaluation, and comparison of peptide and polysaccharide fractions from the same marine species, along with the attempt to correlate the isolated fractions with the anti‐fatigue activity observed in the hydrolysate, remain scarcely reported. Therefore, investigating the anti‐fatigue effects of this multicomponent system is crucial for advancing the application of clamworm resources in the functional food industry. In this study, *Perinereis aibuhitensis* hydrolysate (PAH) was prepared via an optimized enzymatic hydrolysis process, followed by the separation of its polysaccharide and peptide fractions. Comprehensive physicochemical and structural characterizations were performed to identify the key constituents of the hydrolysate. Furthermore, the anti‐fatigue effects of PAH and its separated components were systematically evaluated using rotarod fatigue and exhaustive swimming tests in mice. To elucidate the underlying mechanisms, we further analyzed critical indicators regarding energy metabolism, antioxidant‐associated outcomes, and muscle tissue morphology.

## 2 Material and Methods

### 2.1 Materials and Reagents

Neutrase (200,000 U/g), Flavourzyme (15,000 U/g), Alcalase (200,000 U/g), pepsin (3000–3500 NFU/g), and papain (800,000 U/g) were purchased from Solarbio Science & Technology Co. Ltd. (Beijing, China). American ginseng ( *Panax quinquefolius* ) was purchased from Shandong Shenxiang Chuanqi Biotechnology Co. Ltd. (Weihai, China), with a total ginsenoside content (Rg_1_ + Re + Rb_1_) of approximately 4.54%. Assay kits for blood lactic acid, blood urea nitrogen, creatine kinase, muscle glycogen, liver glycogen, superoxide dismutase (SOD), and glutathione peroxidase (GSH‐Px) were procured from Jiancheng Bioengineering Institute Co. Ltd. (Nanjing, China). All other chemical reagents were of analytical grade and purchased from Sinopharm Chemical Reagent Co. Ltd. (Shanghai, China).

### 2.2 Preparation and Characterization of PAH and Its Polysaccharide and Peptide Fractions

Fresh clamworms (6.0 ± 0.1 g) were purchased from Fujian Yangze Marine Biotechnology Co. Ltd. (Fuzhou, China) and kept frozen at −20°C until use, at which time they were thawed and washed with tap water to remove impurities. The samples were homogenized with ultrapure water at a ratio of 1:3 (w/v). To screen the optimal enzyme, individual proteases (pepsin, alcalase, flavourzyme, neutrase, and papain) were added for hydrolysis under their respective optimal pH and temperature conditions (Table S1) for 4 h. The enzymes were subsequently inactivated by heat treatment at 100°C for 10 min. After cooling to room temperature, the mixture was centrifuged (6000 rpm, 15 min, 4°C), and the supernatant was collected and spray‐dried to obtain PAH. The total protein content in the hydrolysate was determined using the Kjeldahl method, and the total sugar content was determined using the phenol–sulfuric acid method.

PAH was processed using tangential flow filtration with a 30 kDa molecular weight cutoff (MWCO) membrane. The permeate was collected, lyophilized, and designated as the peptide fraction (PAT). The retentate was collected and concentrated via rotary evaporation. The concentrate was precipitated with 3 volumes of 95% (v/v) ethanol. The precipitate was redissolved in water and deproteinized using the Sevage method (chloroform: n‐butanol = 4:1, v/v). This process was repeated five times to ensure the removal of proteins. The supernatant was then dialyzed and lyophilized to obtain the polysaccharide fraction (PAS).

PAS was further fractionated using a DEAE Sepharose Fast Flow anion‐exchange column (Cytiva, Shanghai, China). The crude polysaccharide was dissolved in deionized water and eluted with a linear gradient of NaCl (0, 0.1, 0.3, 0.5, 0.7, and 1.0 M) at a flow rate of 1 mL/min. The eluent was monitored using the phenol–sulfuric acid method. The target fractions were collected, concentrated, dialyzed at 4°C for 48 h, and lyophilized to obtain two subfractions, designated as PAS‐1 and PAS‐2. The samples were mixed with potassium bromide and compressed into pellets for Fourier transform infrared spectroscopy (FTIR) analysis over the range of 4000–400 cm^−1^.

### 2.3 Determination of the Degree of Hydrolysis (DH)

The DH of PAH was determined using the *o*‐phthaldialdehyde (OPA) method (Wei et al. 2024). Samples or standards were reacted with the OPA reagent at 25°C for 2 min, and the absorbance was measured at 340 nm. Serine was used as the standard to calculate the free amino group content. The DH was calculated using Equation (1):

(1) $$\mathrm{DH}\;\left(\%\right)=\frac{h_{1}-h_{0}}{h_{2}-h_{0}}\times 100$$

where $h_{1}$ is the free amino group content in the hydrolysate, $h_{0}$ is the free amino group content in the homogenate, and $h_{2}$ is the free amino group content in the completely hydrolyzed sample.

### 2.4 Determination of Soluble Solid Content

The soluble solid content (ρ) of PAH was determined by the gravimetric method. After centrifugation, the weight of the supernatant was recorded as m_1_. An aliquot (m_2_ grams) of the sample was dried in a pre‐weighed container (m_0_) at 105°C until a constant weight was achieved, with the final weight recorded as m_3_. The yield was calculated using Equation (2):

(2) $$\begin{array}{c} \rho \;\left(\%\right)=\frac{\left(m_{3}-m_{0}\right)\times m_{1}}{m_{2}\times m}\times 100\; \end{array}$$

where m denotes the dry weight of the initial sample.

### 2.5 Molecular Weight Distribution Profiling

The molecular weight distribution of peptides was determined via size‐exclusion chromatography (SEC) with minor modifications to the method described by Zhu et al. (2023). A TSK‐Gel G2000 SWXL column (7.8 mm × 30 cm, TOSOH, Tokyo, Japan) was employed. The mobile phase consisted of acetonitrile/water/trifluoroacetic acid (45:55:0.1, v/v/v) at a flow rate of 0.5 mL/min. Peptides were detected at 214 nm with a column temperature of 30°C. To construct the calibration curve of log MW against retention time, the molecular weight standards used were Gly‐Gly‐Gly (189 Da), Gly‐Gly‐Tyr‐Arg (451 Da), bacitracin (1423 Da), aprotinin (6512 Da), and cytochrome C (12,384 Da).

The molecular weight distribution of polysaccharides was determined using HPLC coupled with an 18‐angle laser light scattering detector (HPLC‐MALS), as described by Zhao et al. (2017). Briefly, the mobile phase was 0.1 M Na_2_SO_4_ at a flow rate of 0.6 mL/min. The column temperature was maintained at 35°C, and the injection volume was 100 μL.

### 2.6 Analysis of Amino Acid Composition

The amino acid composition was analyzed following the method of Li, Ma, et al. (2022), with minor modifications. Briefly, the sample was hydrolyzed with 6 M HCl at 110°C for 22 h. A after cooling, the hydrolysate was diluted to 50 mL with 0.02 M HCl. A 1 mL aliquot of the hydrolysate was dried under a nitrogen stream to remove the acid. Subsequently, it was reconstituted with 2 mL of 0.02 M HCl, filtered through a 0.22 μm membrane, and the total amino acid composition was determined using an automatic amino acid analyzer (LA8080, HITACHI, Tokyo, Japan).

### 2.7 Determination of Monosaccharide Composition

The monosaccharide composition was determined using high‐performance liquid chromatography (HPLC) with pre‐column derivatization by 1‐phenyl‐3‐methyl‐5‐pyrazolone (PMP) (Xie et al. 2020). Briefly, the sample was hydrolyzed with 2 M trifluoroacetic acid (TFA) at 110°C for 4 h. After cooling to room temperature, the acid was removed under a nitrogen stream, and the pH of the hydrolysate was adjusted to approximately 7–8. The neutralized solution was subjected to derivatization. To 10 μL of the hydrolyzed sample, 130 μL of 0.3 M NaOH and 150 μL of PMP derivatization reagent were added. The mixture was incubated in a 70°C water bath in the dark for 30 min. The reaction was terminated and the system neutralized by adding an equal volume of 0.3 M HCl solution. To remove the excess PMP reagent, the derivatized mixture was extracted eight times with 0.5 mL of chloroform. After each extraction, the aqueous phase was carefully collected for subsequent analysis. The samples were analyzed using an Agilent 1260 HPLC system (Santa Clara, CA, USA) equipped with a Poroshell EC‐C18 column maintained at 30°C. The injection volume was 20 μL, the flow rate was 0.4 mL/min, and the detection wavelength was set at 250 nm. Mobile phase A consisted of 0.05 M phosphate buffer (PBS): acetonitrile = 83:17 (v/v), and mobile phase B was 0.05 M PBS: acetonitrile = 65:35 (v/v). The gradient elution program was as follows: 3%–20% B from 0 to 40 min, 20%–100% B from 40 to 41 min, 100% B from 41 to 43 min, a decrease to 3% B from 43 to 44 min, and 3% B from 44 to 46 min.

### 2.8 Animal Experiments

Male C57BL/6 mice (SPF grade, 8 weeks old, 22 ± 2 g) were purchased from Jinan Xingkang Biotechnology Co. Ltd. (Jinan, China) and housed under standard SPF conditions (12 h light/dark cycle, 24°C ± 2°C, 50% relative humidity). The study protocol was approved by the Experimental Animal Ethics Committee of the College of Food Science and Engineering, Ocean University of China (No. OUC‐AE‐2024028). The experimental design is illustrated in Figure 3A. Following a one‐week acclimatization period, mice were randomly divided into five groups (*n* = 10 per group): control group (CON, distilled water), positive control group (PC, ginseng powder, 1000 mg/kg), PA hydrolysate group (PAH, 1000 mg/kg), PA polysaccharide group (PAS, 250 mg/kg), and PA peptide group (PAT, 700 mg/kg). Treatments were administered daily by oral gavage for 30 days. The dosing regimen was designed based on the compositional ratio of PAH, and the PAH dose, when converted based on body surface area, corresponds to an estimated human intake of approximately 80 mg/kg (Reagan‐Shaw et al. 2008).

On day 22, 30 min after administration, mice were placed on a rotarod apparatus rotating at 18 rpm. The time taken for each mouse to fall off the rod due to fatigue was recorded. A mouse that fell off for 3 min was considered fatigued.

On day 25, 30 min after administration, a lead weight (5% of body weight) was attached to the tail of each mouse. The mice were placed in a water bath (25°C, 30 cm depth). The time from the start of swimming until the mouse remained submerged for more than 10 s (exhaustive swimming time) was recorded. No formal a priori sample size calculation was performed. A post hoc power analysis indicated that a sample size of *n* = 10 per group provided > 80% power at α = 0.05 to detect the observed difference in exhaustive swimming time between the control and intervention groups.

On day 30, following a 30 min forced swim, mice were anesthetized, and blood was collected from the orbital sinus. Serum was obtained by centrifugation (3500 rpm, 4°C, 15 min). The liver, spleen, kidneys, pancreas, and gastrocnemius muscles were harvested and weighed. Organ indices were calculated as described by Gao et al. (2018). Serum levels of blood lactic acid, blood urea nitrogen, SOD, GSH‐Px, and creatine kinase, as well as tissue ATP, muscle glycogen, and liver glycogen contents, were measured using commercial assay kits according to the manufacturers' instructions. All animals completed the assessments, and biochemical assays were performed with a minimum of three replicate wells per sample. The left gastrocnemius muscle was fixed in 4% paraformaldehyde for 24 h. Tissues were dehydrated, embedded in paraffin, sectioned, and stained with hematoxylin and eosin (H&E). Coded sections were evaluated blindly under an optical microscope. To quantify the cross‐sectional area of muscle fibers, three random fields were selected and captured under a 10× objective. The clear and complete boundary of each muscle fiber was manually delineated, and the enclosed area was calculated using Image‐Pro Plus 6.0 software.

### 2.9 Statistical Analysis

Data were expressed as mean ± standard deviation (SD). No outliers were excluded from the analysis. The homogeneity of variances was assessed using Levene's test in SPSS version 26.0 (SPSS Inc., Chicago, USA); when *p* > 0.05, equal variances across groups were assumed. Differences were then analyzed using one‐way analysis of variance (ANOVA) followed by Duncan's multiple range test. A *p* value < 0.05 was considered statistically significant. No outliers were excluded from the analysis.

## 3 Results and Discussion

### 3.1 Optimization of Enzymatic Hydrolysis Process

Proteases exhibit markedly different cleavage specificities that directly influence the composition and yield of target products. Thus, selecting an appropriate protease is crucial for optimizing hydrolysis efficiency. In the present study, five common commercial proteases were screened using DH and soluble solid yield as assessment criteria. Both alcalase and flavourzyme exhibited the highest DH and soluble solid content, with no significant difference between them (Figure 1A). Flavourzyme is a complex enzyme system possessing both endoprotease and exopeptidase activities. It can cleave peptide bonds within polypeptide chains and remove specific amino acids from either the N‐ or C‐terminus. This dual action generates a diverse array of peptide fragments, potentially enhancing the flavor profile and solubility of the final product (Chen et al. 2025; Tahir et al. 2024). Although alcalase also demonstrated high hydrolysis efficiency, flavourzyme was selected for further optimization due to its broader specificity and neutral optimal pH, which is advantageous for industrial applications and energy conservation.

**FIGURE 1:**
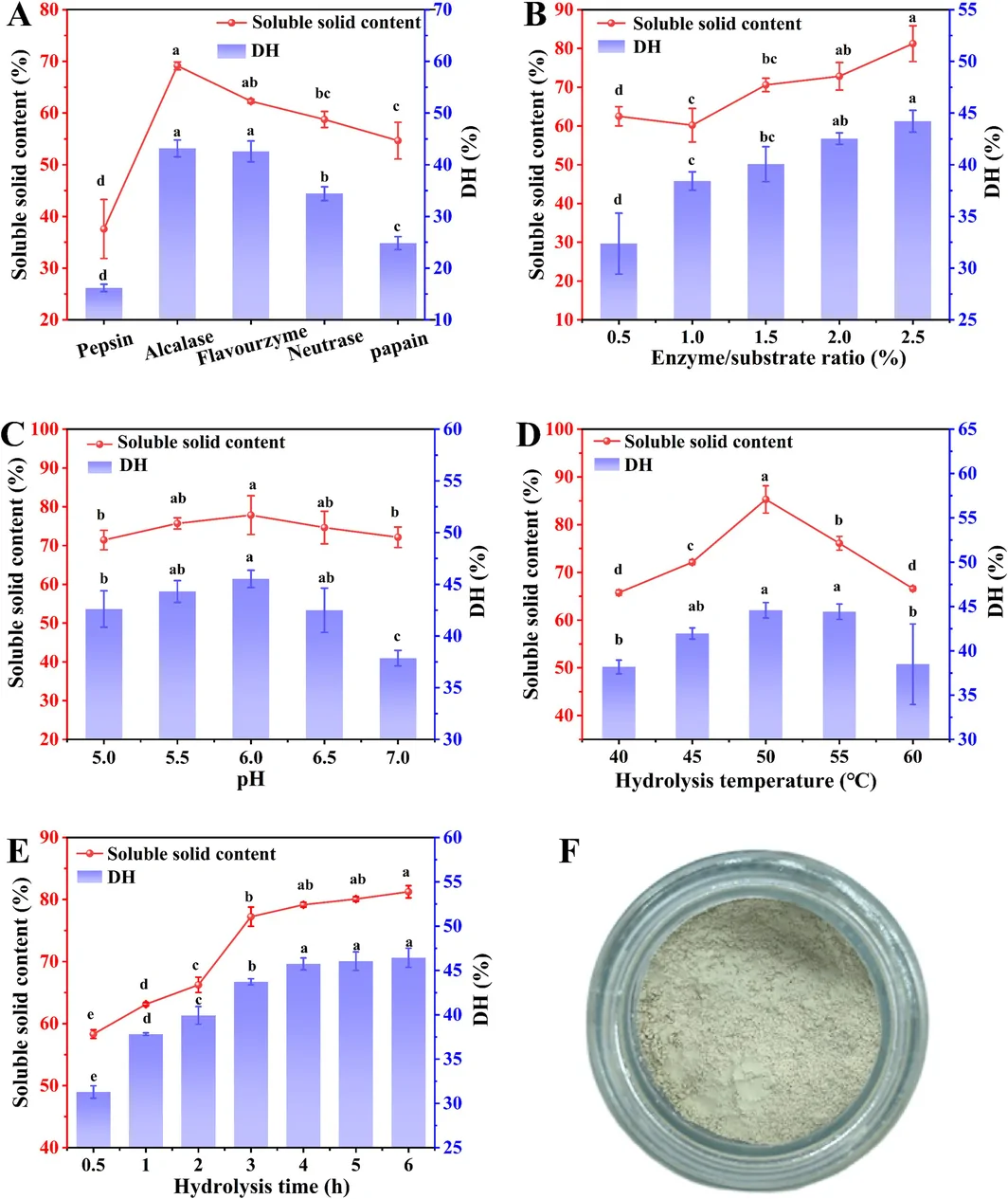
The effects of enzyme type (A), enzyme dosage (B), pH (C), temperature (D), and reaction time (E) on the soluble solids content and degree of hydrolysis of PAH. Data are presented as mean ± SD. Different lowercase letters indicate significant differences (*p* < 0.05). (F) Appearance of spray‐dried powder from the PAH.

A single‐factor experimental design was employed to examine the effects of enzyme concentration, pH, temperature, and hydrolysis time on PAH production. As shown in Figure 1B, both DH and soluble solid content increased with enzyme concentration from 0.5% to 2.5%, approaching a plateau at 1.5%. Further increases in enzyme concentration did not yield significant enhancements. Similar trends were observed for pH, temperature, and hydrolysis time (Figure 1C–E). Consequently, the optimal hydrolysis conditions were established as follows: enzyme concentration of 1.5%, pH 6.0, temperature of 50°C, and a duration of 4 h. Under these optimized conditions, the DH and soluble solid content of PAH reached 45.7% and 83.2%, respectively. The final PAH was spray‐dried into a pale‐yellow powder (Figure 1F) containing 70.71 ± 1.43 g/100 g of protein and 20.13 ± 0.72 g/100 g of total sugar, confirming it as a protein‐rich hydrolysate with a considerable polysaccharide fraction.

### 3.2 Characterization of Peptide and Polysaccharide Fractions

The SEC analysis of peptide molecular weight distribution revealed that PAH contained predominantly small peptides (< 1 kDa, accounting for 77.9% of total peptides), followed by low‐to‐medium molecular weight peptides (1–3 kDa, 13.1%) (Figure 2A). The abundance of low‐molecular‐weight peptides in PAH suggests enhanced bioavailability and potential biological activity (Nwachukwu and Aluko 2019). Previous studies have indicated that hydrolysates rich in small peptides (< 1 kDa) exhibit superior anti‐fatigue effects compared wtih those with higher molecular weights (Zhao et al. 2023). As shown in Figure 2B, PAH possesses a complete amino acid profile comprising 17 amino acids. It is particularly rich in leucine (Leu), glutamic acid (Glu), valine (Val), proline (Pro), and isoleucine (Ile). Notably, PAH has a high content of branched‐chain amino acids (BCAAs: Leu, Ile, Val), totaling 12.28 g/100 g (Table S2). BCAAs are critical for energy supply, stimulating skeletal muscle protein synthesis, and mitigating both central and peripheral fatigue during exercise (Chai et al. 2025). Additionally, Glu modulates basal energy metabolism, while Pro is associated with antioxidant capacity, both of which contribute to improved stamina and fatigue recovery (Miao et al. 2018). These findings support the hypothesis that the peptide component of PAH possesses favorable anti‐fatigue properties.

**FIGURE 2:**
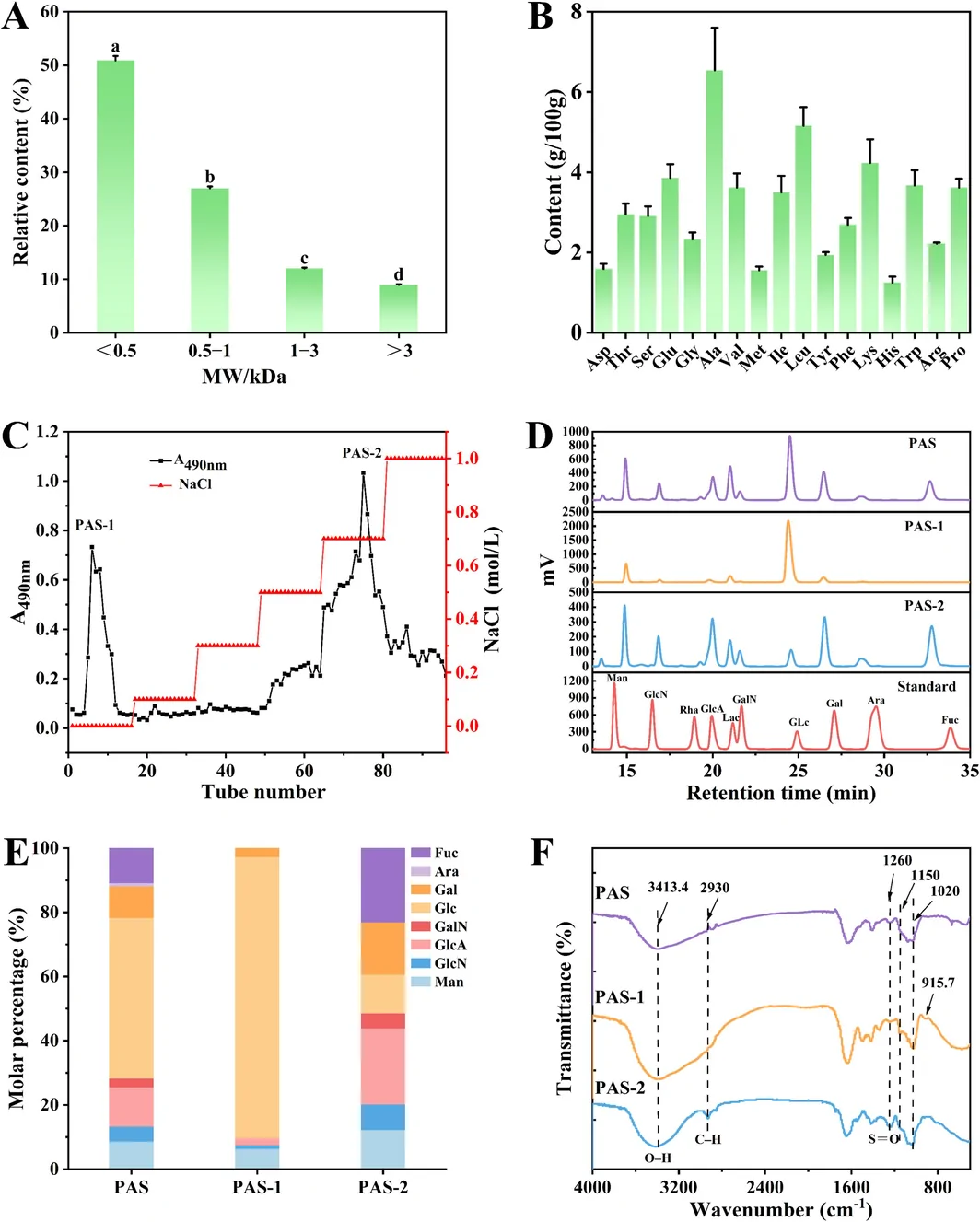
Characterization of peptide and polysaccharide fractions. (A) Molecular weight distribution of peptides. (B) Amino acid composition. (C) Elution profile from the DEAE anion‐exchange column. (D) HPLC chromatogram of monosaccharide composition. (E) Molar percentages of monosaccharides. (F) Fourier‐transform infrared (FTIR) spectrum of PAS. Data are presented as mean ± SD.

The crude polysaccharides (PAS) were further fractionated via DEAE anion‐exchange chromatography, yielding two major fractions, PAS‐1 and PAS‐2, with recovery rates of 16.6% and 39.2%, respectively (Figure 2C). As determined by SEC‐MALS, the weight‐average molecular weights were 1.022 × 10^3^ kDa for PAS‐1 and 6.678 × 10^2^ kDa for PAS‐2 (Table 1). Figure 2D,E presents the monosaccharide composition of PAS and its purified fractions. The primary constituents of PAS were glucose (Glc, 50.05%), glucuronic acid (GlcA, 12.22%), fucose (Fuc, 10.81%), galactose (Gal, 9.73%), and mannose (Man, 8.59%). PAS‐1 was predominantly composed of Glc (90.07%), with minor amounts of Man, GlcA, and Gal. Based on its monosaccharide profile and high molecular weight, PAS‐1, with structural features suggestive of glycogen, is speculated to serve as an energy reserve (Bouhedi et al. 2021; Durou et al. 2005). In contrast, PAS‐2 was primarily composed of GlcA (23.68%), Fuc (23.11%), and Gal (16.27%), suggesting that PAS‐2 is tentatively identified as a sulfated heteroglycan rich in glucuronic acid and fucose. FTIR spectra confirmed typical polysaccharide features (Figure 2F): a broad band at ~3413 cm^−1^ (O–H stretch), a peak at ~2926 cm^−1^ (C–H stretch), and a region between 950 and 1200 cm^−1^ (C–O/C–C pyranose ring vibrations). The intense absorption peaks at around 1150 cm^−1^ (often attributed to the α‐1,4, C–O–C glycosidic bond) and 1022 cm^−1^ (often attributed to the anhydro glucose ring in disordered arrangements) are characteristic FTIR bands used for glycogen detection in animal cells and tissues (Nikonenko et al. 2005; Van Soest et al. 1995; Wood et al. 1996), supporting the tentative assignment of PAS‐1 as a glycogen‐like glucans. A characteristic S=O stretching vibration at ~1260 cm^−1^ in PAS and PAS‐2 suggests the presence of sulfate groups (Gómez‐Ordóñez and Rupérez 2011), which is consistent with the monosaccharide analysis indicative of putative sulfated heteroglycans.

**TABLE 1:** The molecular weight of clamworm purified polysaccharides.

Polysaccharide component	Number‐average molecular weight (*M*_*n*_)/kDa	Weight‐average molecular weight (*M*_*w*_)/kDa	*M*_*w*_/*M*_*n*_
PAS‐1	7.420 × 10^2^ (±0.963%)	1.022 × 10^3^ (±0.833%)	1.377 (±0.872%)
PAS‐2	4.565 × 10^2^ (±0.872%)	6.678 × 10^2^ (±0.479%)	1.463 (±0.995%)

### 3.3 Effects of PAH Supplementation on Exercise Capacity in Mice

Body weight changes serve as indicators of physiological status, while organ indices are used to assess potential toxicity. Throughout the administration period, all mice exhibited a progressive increase in body weight with no significant inter‐group differences (*p* > 0.05) (Figure 3B). A slight weight decrease observed in the CON group after day 25 may be attributed to physical exertion from the rotarod test. Furthermore, no significant differences were observed in the organ indices (heart, liver, spleen, and kidney) among the groups (*p* > 0.05) (Figure 3C). These results indicate that PAH, PAS, and PAT interventions had no adverse effects on the general health or growth of the mice.

**FIGURE 3:**
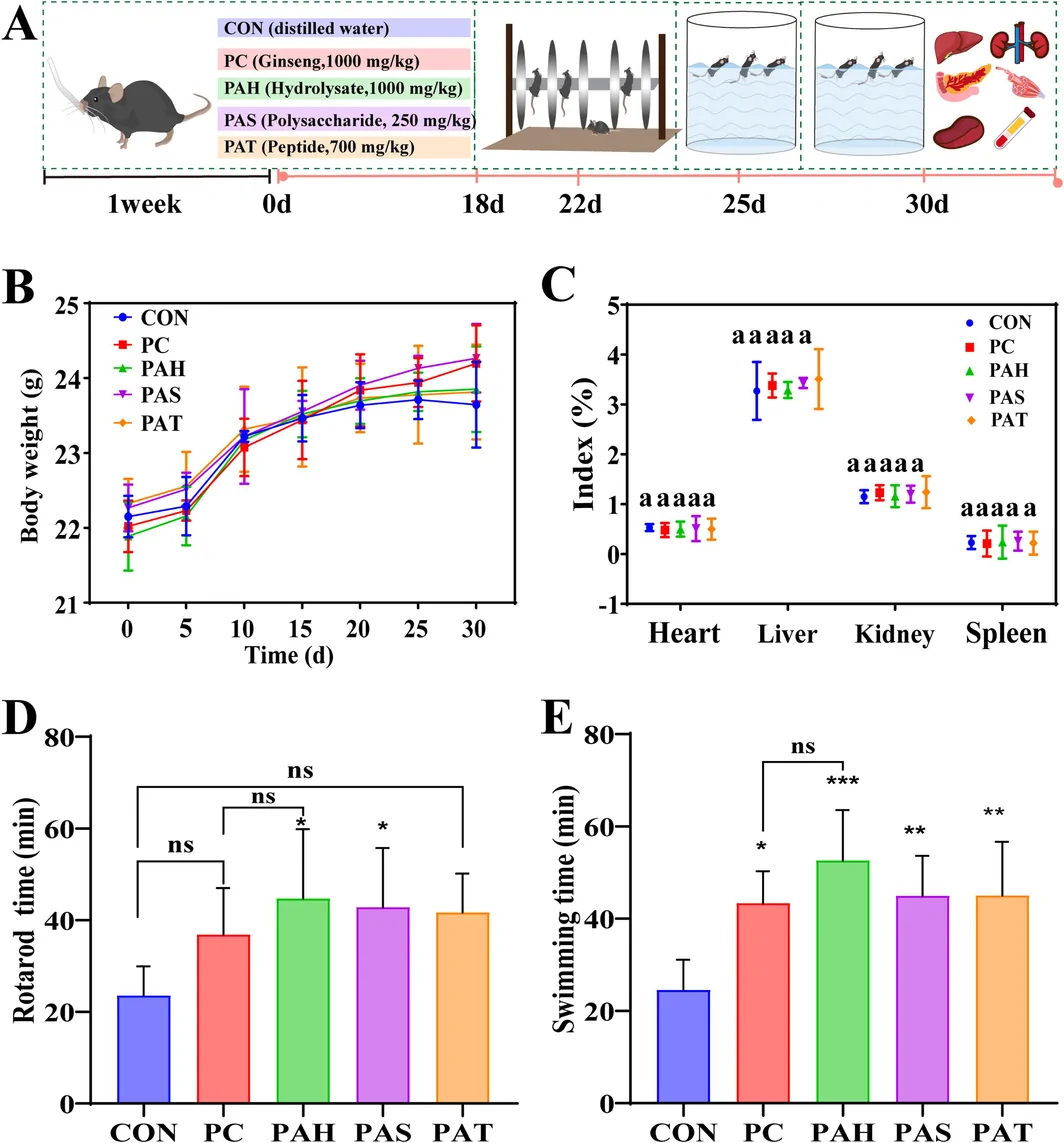
(A) Experimental design and timeline of the exercise test in mice; “0 day” indicates the end of the acclimation period. Effects of PAH on body weight (B), organ indices (C), rotarod retention time (D), and loaded swimming time (E) in mice. Data are presented as mean ± SD. Ns, not significant between groups. Compared with the CON group, *p < 0.05, **p < 0.01, ***p < 0.001.

The most apparent manifestation of physical fatigue is decreased exercise endurance; therefore, a rotarod test was employed to evaluate the exercise endurance of mice after 22 days of gavage. As shown in Figure 3D, the rotarod retention time was significantly prolonged in the PAH and PAT groups compared with the CON group. These results demonstrate that intervention with PAH and PAT can significantly enhance the exercise endurance of mice.

The exhaustive swimming test is a standard method for evaluating anti‐fatigue efficacy. On day 25, the exhaustive swimming time was significantly prolonged in all treatment groups (PAH, PAS, PAT) and the positive control (PC, ginseng) group compared with the CON group (*p* < 0.05) (Figure 3E). Specifically, PAH, PAS, and PAT prolonged the swimming time by 83.24%, 83.58%, and 114.52%, respectively. These findings confirm that the hydrolysate and its separated components (polysaccharides and peptides) all possess significant anti‐fatigue activity.

### 3.4 Effect of PAH on Muscle Damage in Exercised Mice

Strenuous physical exertion has been shown to cause disruption of muscle fibers and injury to muscle tissue. Histopathological examination of the gastrocnemius muscle was performed following a 30 min swimming test (Figure 4A). Muscle cells in the CON group exhibited a markedly disorganized arrangement, shrinkage, and enlarged intercellular spaces indicating that the tissue architecture of the mouse gastrocnemius muscle was impaired. In contrast, the PC group showed well‐preserved muscle cell morphology with compact and dense alignment. Compared with the CON group, the intervention groups (PAH, PAS, and PAT) displayed improved organization of muscle fibers. These results indicate that supplementation with PAH, PAS, and PAT can effectively ameliorate exercise‐induced damage to the gastrocnemius muscle caused by excessive exercise.

**FIGURE 4:**
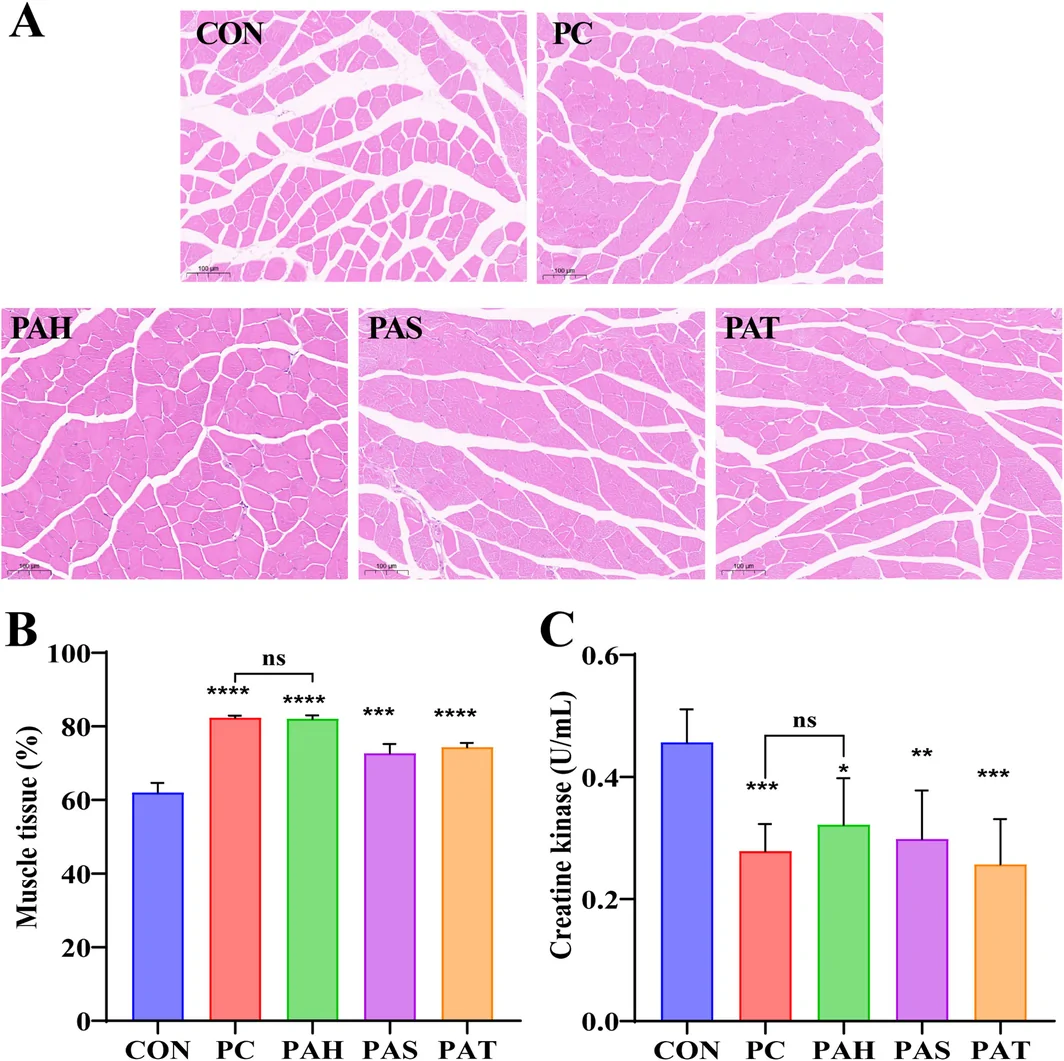
Effect of sandworm PAH on muscle damage in exercised mice. (A) H&E staining of the gastrocnemius muscle, scale bar = 100 μm. (B) Relative cross‐sectional area of gastrocnemius muscle fibers. (C) Serum creatine kinase content. Data are presented as mean ± SD. Ns, not significant between groups. Compared with the CON group, *p < 0.05, **p < 0.01, ***p < 0.001.

For quantitative evaluation of muscle morphology, the relative cross‐sectional area occupied by muscle fibers in the murine gastrocnemius was quantified (Figure 4B). Compared with the CON group, the muscle fiber proportions in the intervention groups (PAH, PAS, and PAT) were significantly increased by 32.26%, 17.20%, and 19.89%, respectively (*p <* 0.05). These quantitative results confirm that all three interventions confer a certain degree of protection against intensive exercise‐induced muscle damage.

Creatine kinase is predominantly localized in muscle tissues, where it functions to phosphorylate creatine to rapidly supply energy to cells (Darabedian et al. 2023). Prolonged exercise can compromise cell membrane integrity, increasing permeability and leading to the release of creatine kinase into the bloodstream. Therefore, elevated serum creatine kinase levels serve as a biomarker of muscle injury. As shown in Figure 4C, all intervention groups (PAH, PAS and PAT) exhibited significantly reduced serum creatine kinase levels compared with the CON group (0.46 ± 0.05 U/mL), with reduction rates of 29.56%, 34.67%, and 43.80%, respectively (*p <* 0.05). These results indicate that PAH, PAS, and PAT administration mitigates exercise‐induced muscle injury in mice by inhibiting the leakage of intracellular creatine kinase into the bloodstream.

### 3.5 Effect of PAH on Energy Metabolism and Oxidative Stress in Exercised Mice

Energy depletion and metabolite accumulation are primary causes of fatigue (Li, Xie, et al. 2024). High‐intensity exercise leads to blood lactic acid accumulation, which lowers intramuscular pH and inhibits ATP synthesis (Zhou et al. 2023). As shown in Figure 5A, after the swimming test, the blood lactic acid level in the CON group (5.13 ± 0.55 mmol/L) was significantly higher than that in the PC group (3.41 ± 0.62 mmol/L). Compared with the CON group, the blood lactic acid levels in the PAH, PAS, and PAT groups were significantly reduced by 18.01%, 30.98%, and 20.97%, respectively (*p* < 0.05). These results indicate that PAH can effectively reduce blood lactic acid accumulation following fatiguing exercise.

**FIGURE 5:**
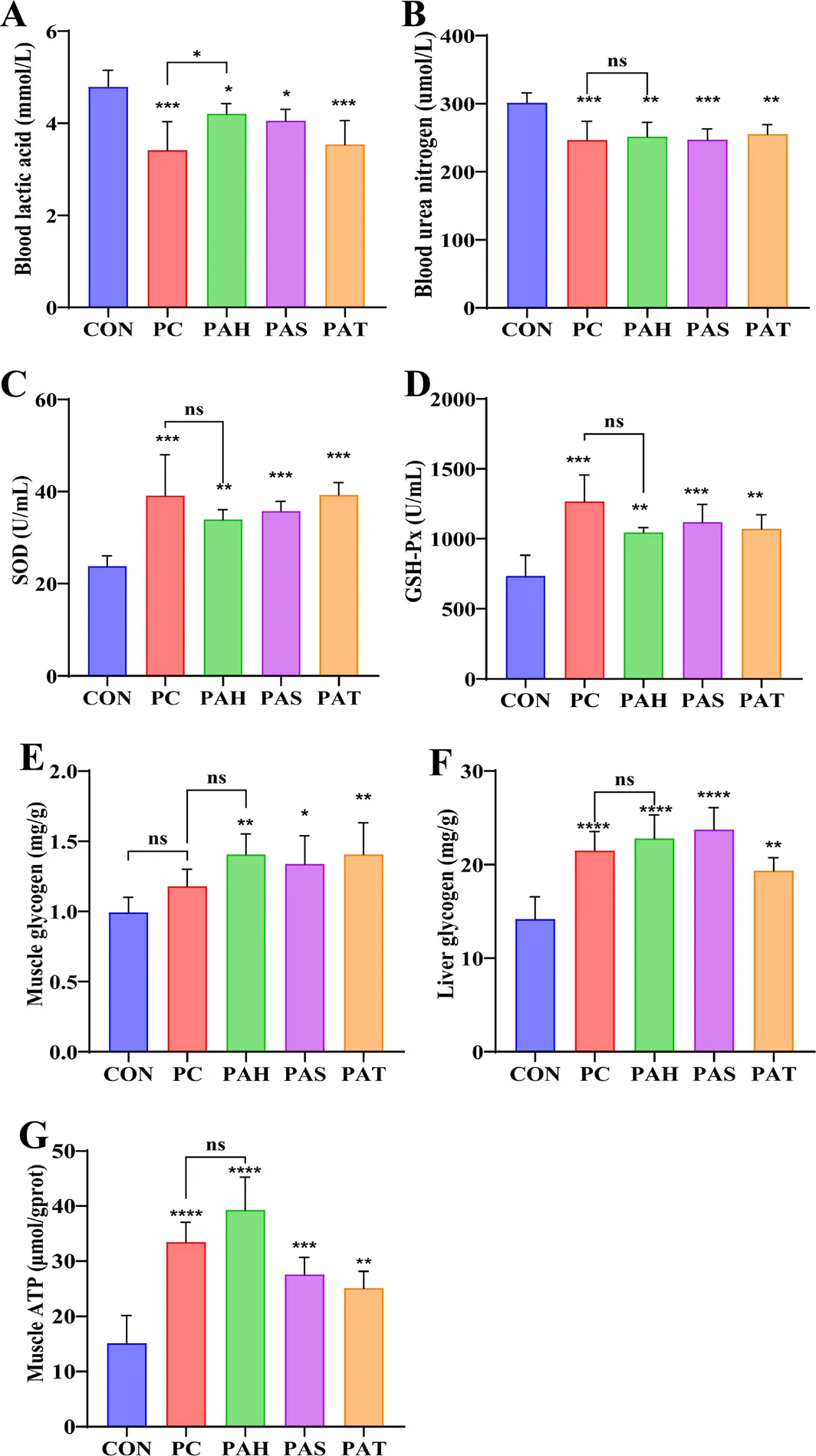
Effect of PAH on energy metabolism and antioxidant‐associated outcomes in exercised mice. (A) Blood lactic acid (BLA); (B) Urea nitrogen (BUN); (C) SOD; (D) GSH‐px; (E) Muscle glycogen (MG); (F) Liver glycogen (LG); (G) ATP. Data are presented as mean ± SD. “ns,” not significant between groups. Compared with the CON group, *p < 0.05**p < 0.01, ***p < 0.001.

Blood urea nitrogen serves as a highly sensitive fatigue marker, and its elevation typically stems from accelerated protein catabolism (Zhou et al. 2025). As illustrated in Figure 5B, blood urea nitrogen levels were significantly lower in the PAH, PAS, and PAT groups compared with the CON group (*p* < 0.05), with reductions of 16.53%, 17.99%, and 15.33%, respectively. Notably, the effects observed in the PAH, PAS, and PAT groups were comparable to those of the PC group. These results demonstrate that PAH effectively alleviated exercise‐induced blood urea nitrogen accumulation, thereby facilitating recovery from physical fatigue.

Intense exercise induces oxidative stress through excessive ROS production. Antioxidant enzymes such as SOD and GSH‐Px are vital for scavenging these radicals, thereby alleviating physical fatigue. As shown in Figure 5C,D, serum SOD and GSH‐Px activities were significantly elevated in the PAH, PAS, and PAT groups compared with the CON group (*p* < 0.05). Specifically, SOD activity increased by 42.92%–65.34%, and GSH‐Px activity increased by 42.51%–52.49%. These results suggest that PAH and its components may contribute to enhancing the endogenous antioxidant defense system. However, it should be noted that only two antioxidant enzymes were evaluated in this study. A more comprehensive assessment, including additional markers such as MDA, ROS, or CAT, would be necessary to fully characterize the antioxidant capacity of PAH.

Muscle glycogen and liver glycogen serve as crucial energy sources during sustained physical activity, and their reserve levels are indicative of anti‐fatigue capacity (Zhao et al. 2024). ATP functions as the immediate energy currency for all physiological activities and is crucial for alleviating physical fatigue (Cai et al. 2023). To assess the capacity of PAH to retard energy exhaustion in mice, the contents of energy substrates (muscle glycogen, liver glycogen, and ATP) were measured after 30 min of swimming (Figure 5E–G). Compared with the CON group, the muscle glycogen levels in the PAH, PAS, and PAT groups increased by 41.68%, 34.79%, and 41.68%, respectively; liver glycogen content was significantly elevated by 11.55, 13.71, and 7.24 mg/g, respectively; meanwhile, ATP levels also rose significantly by 160.02%, 82.49%, and 66.11%, respectively (*p <* 0.05). Notably, no significant difference in glycogen and ATP levels was observed between the PC and PAH groups. These results indicate that the anti‐fatigue effect of PAH may be attributed to enhanced glycogen storage and improved energy metabolism.

### 3.6 Correlation Analysis and Discussion

Pearson correlation analysis was performed to examine the relationships between behavioral and biochemical indicators (Figure 6). Rotarod endurance and exhaustive swimming time exhibited significant positive correlations with muscle glycogen and ATP levels, as well as with SOD and GSH‐Px activities, but were negatively correlated with serum creatine kinase activity and the concentrations of two fatigue metabolites, namely blood lactic acid and blood urea nitrogen. These results indicate a significant association among energy substrate levels, fatigue metabolite concentrations, antioxidant enzyme activities, and exercise endurance in mice. Significant negative correlations were also observed between SOD and GSH‐Px activities and both creatine kinase activity and blood lactic acid/urea nitrogen (BLA/BUN) concentrations. These findings suggest a potential relationship between the anti‐fatigue effects of PAH and the observed enhanced antioxidant enzyme activities. It is plausible that improvements in antioxidant enzyme activities may be associated with reduced creatine kinase efflux from muscle cells and enhanced energy metabolism, which together may contribute to the enhancement of exercise endurance in mice.

**FIGURE 6:**
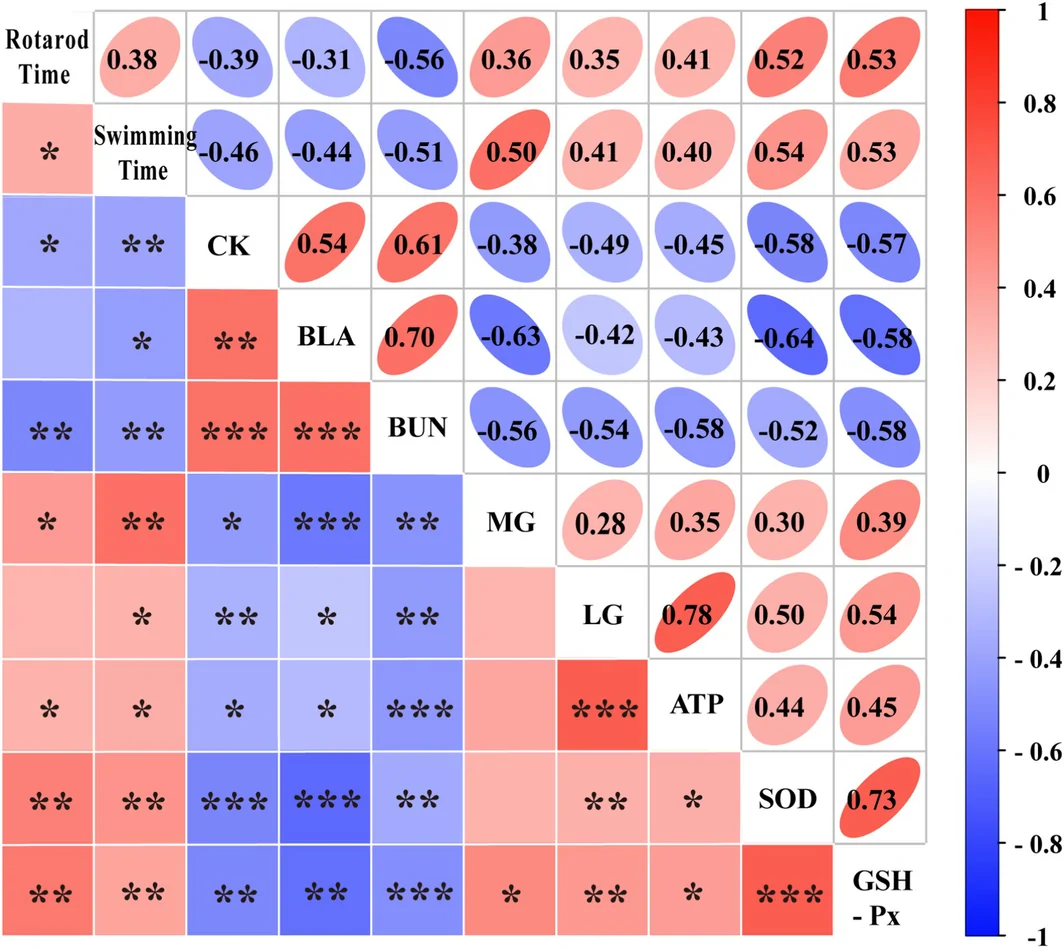
Pearson correlation analysis among exercise capacity, metabolite accumulation, energy storage, muscle damage, and oxidative stress. Red indicates a positive correlation, while blue indicates a negative correlation. Significant correlations are indicated by *p < 0.05, **p < 0.01, ***p < 0.001.

The potent anti‐fatigue efficacy of PAH may be associated with its specific peptide composition, particularly the high abundance of BCAAs, which account for 12.28% of the total amino acids (Figure 2B; Table S2). Unlike other amino acids that are metabolized primarily in the liver, BCAAs are oxidized in skeletal muscle to serve as a direct energy source during endurance exercise. Although not directly investigated in this study, the anti‐fatigue mechanism of BCAAs has been explained in the literature by the “Central Fatigue Hypothesis” (Blomstrand 2011). According to this hypothesis, BCAAs compete with free tryptophan for transport across the blood–brain barrier via the L‐type amino acid transporter. It has been proposed that elevated plasma BCAA levels competitively inhibit the cerebral uptake of tryptophan, thereby reducing the synthesis of serotonin, a neurotransmitter known to induce drowsiness and central fatigue (Sujean et al. 2013). However, further studies involving the measurement of serotonin, tryptophan transport, or brain neurotransmitters are required to validate this hypothesis.

Additionally, leucine, a key component of PAT, is a well‐established activator of the mTOR signaling pathway (Zheng et al. 2019), which promotes muscle protein synthesis and mitigates exercise‐induced muscle damage. This is consistent with the observed reductions in serum creatine kinase and blood urea nitrogen levels in this present study, although the activation of mTOR‐related proteins (e.g., p‐mTOR, p70S6K) was not directly assessed. Therefore, the current data support the physiological anti‐fatigue effects of PAH and its peptide fraction, while the proposed molecular mechanisms remain hypothetical and await direct experimental validation in future studies.

PAS may contribute to the anti‐fatigue activity through distinct structural mechanisms driven by its polysaccharide components, which display structural features suggestive of glycogen and sulfated heteroglycans. The glycogen‐like PAS‐1 may function by optimizing glucose metabolism and upregulating glycogen synthase activity, thereby enhancing hepatic and muscle glycogen reserves (Hao et al. 2014). More importantly, the putative sulfated heteroglycans (PAS‐2) could exhibit significant antioxidant capacity due to the presence of sulfate groups, which may act as effective hydrogen donors to scavenge free radicals. Although not directly investigated in this study, this antioxidant defense may be associated with the activation of the NRF2/HO‐1 signaling pathway, as reported for other sulfated polysaccharides (Luo et al. 2023). It has been demonstrated that upon activation by sulfated polysaccharides, NRF2 translocates to the nucleus and binds to the Antioxidant Response Element (ARE), thereby upregulating downstream enzymes such as SOD and GSH‐Px. This mechanism could potentially clear the “oxidative surge” triggered by exhaustive exercise, protecting mitochondrial integrity and delaying the onset of oxidative stress‐induced fatigue. However, it must be emphasized that the involvement of the NRF2/HO‐1 pathway in the anti‐fatigue effects observed in this study remains a hypothesis based on the literature and requires direct experimental validation in future work.

In summary, although this study identified both peptides and polysaccharides as the primary bioactive constituents responsible for the anti‐fatigue activity of PAH at a fixed dose, the single‐dose design has inherent limitations in elucidating the dose–effect relationship and the minimum effective dose threshold of PAT. Future studies should incorporate multiple dose gradients of PAT to systematically evaluate the dose‐dependent anti‐fatigue effects, thereby providing a more precise dosage reference for subsequent clinical translation and functional product development.

## 4 Conclusion

In this study, PAH was successfully prepared under optimized enzymatic conditions (1.5% Flavourzyme, pH 6.0, 50°C, 4 h), achieving a degree of hydrolysis of 45.7% and a soluble solid content of 83.2%. In vivo evaluations demonstrated that PAH, along with its isolated peptide and polysaccharide fractions, significantly prolonged rotarod retention and exhaustive swimming times in mice. Structural characterization revealed that the bioactivity of PAH may be attributed to its multicomponent composition: (1) high‐abundance small peptides rich in BCAAs, which may contribute to alleviating central fatigue and promoting muscle protein synthesis; (2) putative glycogen‐like polysaccharides (PAS‐1) that could potentially enhance hepatic and muscle energy reserves; and (3) tentative sulfated heteroglycans (PAS‐2) that may be associated with the observed improvements in antioxidant enzyme activities (SOD, GSH‐Px), suggesting a potential role in counteracting oxidative stress. Physiological analyses demonstrated that PAH supplementation effectively regulated energy metabolism by increasing ATP and glycogen storage while simultaneously reducing the accumulation of harmful metabolites and preventing exercise‐induced muscle damage. Collectively, these results suggest that PAH may exert its anti‐fatigue effects through multiple pathways, including energy replenishment, metabolic regulation, and antioxidant enhancement. Therefore, PAH holds significant potential as a natural, high‐value functional ingredient for fatigue recovery and exercise performance enhancement.

## Author Contributions

**Dan Qiu:** writing – original draft, software. **Songran Fang:** investigation. **Fengying Cui:** investigation. **Honglei Zhou:** methodology, resources. **Meichao Zhang:** visualization. **Zifang Zhao:** resources. **Wenhai She:** methodology. **Adewale Olusegun Obadina:** conceptualization. **Haohao Wu:** writing – review and editing, supervision. **Wei Wang:** writing – review and editing, supervision, methodology.

## Funding

This study was financially supported by Hainan Province's Key Research and Development Project (No. ZDYF2024XDNY191), Major Science and Technology Project of Haikou City (No. 2023‐001 and 2025‐004), and National Natural Science Foundation of China (No. 32272240).

## Conflicts of Interest

The clam raw materials used in this study were provided by Fujian Yangze Marine Biotechnology Co. Ltd. Honglei Zhou is an employee of this company, which is also a collaborative partner in this research. The authors declare that they have no known competing financial interests or personal relationships that could have appeared to influence the work reported in this paper.

## Supporting information


**Table S1:** The optimal reaction condition of five proteases.
**Table S2:** Amino acid composition of clamworm hydrolysate.

## Data Availability

The data that support the findings of this study are available on request from the corresponding author. The data are not publicly available due to privacy or ethical restrictions.
