# Supplementary material for: Preparation, Characterization, and Anti‐Fatigue Activity of Clamworm (Perinereis aibuhitensis) Hydrolysate and Its Peptide and Polysaccharide Fractions
Source: Food Sci Nutr. 2026 Nov 11;14(11):e72319. doi: 10.1002/fsn3.72319 (PMC13753079; doi:10.1002/fsn3.72319)
Supplement: Supplementary file 1 [file FSN3-14-e72319-s001.docx]

**Supplementary data**

**Table S1** The optimal reaction condition of five proteases

| Type of enzyme | Temperature (°C) | pH |
| --- | --- | --- |
| Neutrase | 50 | 7.0 |
| Flavourzyme | 50 | 7.0 |
| Alcalase | 55 | 9.5 |
| Pepsin | 40 | 2.5 |
| papain | 55 | 7.0 |

**Table S2** Amino acid composition of clamworm hydrolysate

| Amino acid | Concentration in PAH samples (g/100 g) |
| --- | --- |
| Asp | 1.59±0.13 |
| Thr | 2.95±0.27 |
| Ser | 2.91±0.24 |
| Glu | 3.86±0.34 |
| Gly | 2.33±0.17 |
| Ala | 6.54±1.06 |
| Val* | 3.62±0.35 |
| Met | 1.56±0.09 |
| Ile* | 3.5±0.41 |
| Leu* | 5.16±0.46 |
| Tyr | 1.94±0.07 |
| Phe | 2.69±0.17 |
| Lys | 4.23±0.59 |
| His | 1.25±0.15 |
| Trp | 3.67±0.38 |
| Arg | 2.22±0.03 |
| Pro | 3.62±0.22 |
| BCAA | 12.28±0.52 |
| Total | 53.62±1.81 |
